# The Impact of Clinical Factors, ALK Fusion Variants, and BIM Polymorphism on Crizotinib-Treated Advanced EML4–ALK Rearranged Non-small Cell Lung Cancer

**DOI:** 10.3389/fonc.2019.00880

**Published:** 2019-09-23

**Authors:** Yen-Ting Lin, Yi-Nan Liu, Jin-Yuan Shih

**Affiliations:** ^1^Graduate Institute of Clinical Medicine, College of Medicine, National Taiwan University, Taipei, Taiwan; ^2^Department of Internal Medicine, National Taiwan University Hospital and College of Medicine, National Taiwan University, Taipei, Taiwan

**Keywords:** non-small cell lung cancer, ALK, ALK variant, BIM, crizotinib

## Abstract

Patients' clinical factors and genetics factors such as anaplastic lymphoma kinase (ALK) fusion variants and BIM (Bcl-2-like 11) polymorphism were reported to be associated with clinical outcome in crizotinib-treated advanced non-small cell lung cancer (NSCLC). However, the results were still controversial. We analyzed outcome of 54 patients with known ALK fusion variants who received crizotinib for advanced NSCLC. Thirty of them had successful BIM polymorphism analysis and 6 (20%) had a BIM deletion. Multivariate Cox regression analysis found that previous anticancer therapy [adjusted hazard ratio (aHR) 1.35, 95% confidence interval (CI), 1.04–1.76 for each additional line of therapy, *p* = 0.025] and Eastern Cooperative Oncology Group (ECOG) performance status ≥2 (aHR 8.35, 95% CI, 1.52–45.94, *p* = 0.015) were independent factors for progression-free survival (PFS). Only ECOG performance status ≥2 (aHR 7.20, 95% CI, 1.27–40.79, *p* = 0.026) was an independent factor for overall survival (OS). Neither ALK fusion variants nor the presence of a BIM deletion was associated with crizotinib PFS or OS. After adjusting with clinical factors, different ALK variants and BIM polymorphism might not be independent factors for crizotinib PFS or OS in advanced NSCLC with ALK rearrangement.

## Introduction

In 2007, the echinoderm microtubule-associated protein-like 4 (EML4)–anaplastic lymphoma kinase (ALK) gene rearrangement was first discovered as a driver oncogene for non-small cell lung cancer (NSCLC) (1). Inversion in chromosome 2p fused the N-terminal domain of EML4 to the intracellular kinase domain of ALK, causing constitutive activation of tyrosine kinase, leading to uncontrolled cell growth and proliferation. During the following 10 years, targeting ALK with tyrosine kinase inhibitors (TKIs) has achieved great success. The first-generation ALK TKI crizotinib had better progression-free survival (PFS) (10.9 vs. 7.0 months) and a better overall response rate (ORR) (74 vs. 45%) than chemotherapy in treating naïve ALK rearranged {ALK positive [ALK(+)]} NSCLC in the PROFILE 1014 study (2). Crizotinib has been approved by the US Food and Drug Administration (US FDA) as first-line treatment for ALK(+) advanced NSCLC (3). The second-generation ALK TKIs alectinib (CH5424802/ RO5424802) and ceritinib (LDK378) also showed promising activity in controlling ALK(+) NSCLC in phase 3 trials (4, 5). Moreover, in ALK TKI-naïve patients treated with brigatinib, a next-generation ALK TKI, the PFS was longer than patients treated with crizotinib (6). The development of ALK TKI for use against ALK(+) NSCLC is one of the best stories in the history of developing anticancer therapy.

However, most patients still experienced disease progression after ALK TKI treatment. The latest released data for East Asian patients in PROFILE 1029 revealed that median PFS was 11.1 (95% confidence interval, CI: 8.3–12.6) months for first-line crizotinib-treated advanced ALK(+) NSCLC patients (7). Several factors were reported to be associated with crizotinib PFS, but the two main groups were clinical factors and genetic factors. Traditional clinical factors such as the patient's performance status (8, 9) and brain metastasis (10–12) prior to crizotinib treatment were reported to influence crizotinib PFS. Among the genetic factors, one of the most common was ALK fusion variants. In the preclinical data, different ALK fusion variants were associated with crizotinib sensitivity. ALK fusion variant 2 had lower crizotinib IC_50_ than variant 3. Longer ALK fusion variants were the most unstable and were supposed to be more sensitive to crizotinib than shorter ALK fusion variants (13). The presence of a tandem atypical beta-propeller in the EML protein (TAPE) domain was reported to influence the stability of EML4–ALK protein (14); “short variants,” such as variants 3a/b and 5a/b, lack a TAPE domain (15) and might be less responsive to crizotinib than the longer TAPE-containing variants, such as variant 1 and variant 2 (16). A circular RNA F-circEA found in only variant 3 was reported to promote cancer cell migration and proliferation (17). However, in spite of the supposed mechanism, the real-world data were conflicting. ALK variant 1 (18), variant 2 (19), and variants other than variant 3 (16, 20) were reported to have a better crizotinib PFS, but there were also several reports indicating that all variants had a similar outcome (21, 22). In fact, the largest cohort to date reported there was no crizotinib PFS difference between variant 1 and variant 3 (23). Whether or not different EML4–ALK fusion variants influence crizotinib PFS remains controversial.

Another interesting genetic factor was Bcl-2-like 11 (BIM). BIM is a pro-apoptotic member of the B-cell CLL/lymphoma 2 (BCL2) family of proteins, discovered in Asia only. Its upregulation is required for TKIs to induce apoptosis in kinase-driven cancers (24). The BIM deletion polymorphism was reported to be associated with primary resistance to or a short PFS with epidermal growth factor receptor (EGFR) TKI in advanced EGFR-mutant NSCLC (24, 25). Another report indicated that BIM deletion was related to a poor crizotinib response in advanced ALK(+) NSCLC (26). However, in our previous study, we could not find a relationship between the BIM deletion polymorphism and primary EGFR TKI resistance among our 327 Taiwanese patients, while 52 (16%) of them were positive for BIM deletion (27). In this study, we aimed to analyze the association of clinical factors and genetic factors, including ALK fusion variants and BIM polymorphism, with crizotinib PFS and overall survival (OS) in advanced EML4–ALK(+) NSCLC patients.

## Methods

### Patients

This study retrospectively enrolled patients receiving crizotinib for EML4–ALK rearrangement stage IV or postoperative recurrent (advanced) NSCLC between December, 31, 2010, and December, 31, 2017, at the National Taiwan University Hospital. Only patients with data on EML4–ALK variants using reverse transcription polymerase chain reaction (RT-PCR) were included. Patients who stopped crizotinib within 30 days due to intolerable side effects were excluded. Patients' baseline characteristics, including age, gender, smoking status, previous anticancer therapy, Eastern Cooperative Oncology Group (ECOG) performance status (28), prior brain metastasis, EML4–ALK variants, and status of BIM polymorphism, were checked. The patients were treated and followed up based on the clinician's decision. A blinded chest physician who was not involved in patient management and did not know the laboratory data on EML4–ALK variants and BIM polymorphism retrospectively reviewed the chart and images to determine disease progression according to RECIST criteria version 1.1 (29). PFS was defined as the duration from the first dose of crizotinib to disease progression or death during treatment. OS was defined as the duration from the first dose of crizotinib to the patient's death. Each patient's best overall response, PFS, and OS were recorded. This study was approved by the Institutional Review Board of National Taiwan University Hospital. Written informed consent was obtained from all patients before checking their cancer specimens for molecular studies. All methods were performed in accordance with the relevant guidelines and regulations.

### Analysis for EML4–ALK Fusion Gene

Using immunohistochemistry (IHC) stain, we checked the patients' cancer specimens for ALK using Ventana ALK (D5F3) antibody. We further analyzed cancer specimens for EML4–ALK variants using RNA RT-PCR, as previously described (30). In brief, RNA extracted from patients' tissue specimens were collected for RT-PCR amplification by a OneStep RT-PCR Kit (Qiagen) using the following primers: 5′-TGGCTGATGTTTTGAGGCGT-3′ (forward, on exon 2 of EML4), 5′-AGAGCCCACACCTGGGAAAG-3′ (forward, on exon 13 of EML4), 5′-CCACACAGACGGGAATGAAC-3′ (forward, on exon 18 of EML4), and 5′-AGCAAAGCAGTAGTTGGGGT-3′ (reverse, on exon 20 of ALK). The PCR conditions were as follows: 50°C for 30 min, 95°C for 15 min (94°C for 50 s, 60°C for 50 s, 72°C for 60 s) × 40 cycles, and 72°C for 10 min. RT-PCR amplicons were purified and sequenced with Sanger sequencing in both sense and antisense directions. Because the length of the ALK fusion protein may contribute to its stability (13) and probably crizotinib PFS, we also separated different ALK fusion variants into a long group and a short group. Short ALK fusion variants were defined as variants that do not have the TAPE main, i.e., variant 3a, variant 3b, variant 5a, and variant 5b. Long ALK fusion variants were defined as EML4–ALK fusion variants that contain the TAPE, i.e., all variants other than variant 3a, 3b, 5a, or 5b (15, 22).

### Analysis for the BIM Polymorphism

We checked patient cancer specimens with known EML4–ALK fusion variants for further BIM polymorphism analysis, as previously described (24). Cancer DNA was extracted from cancer specimens using the QIAamp DNA Mini Kit (Qiagen). PCR reactions were done to determine the presence of wild-type or deletion alleles using high-fidelity JumpStart™ REDAccuTaq® LA DNA Polymerase (Sigma) with the following conditions: 96°C for 30 s (94°C for 15 s, 60°C for 60 s, 68°C for 10 min) × 30 cycles, and 68°C for 20 min. The forward primer was 5′-AATACCACAGAGGCCCACAG-3′ and the reverse primer was 5′-GCCTGAAGGTGCTGAGAAAG-3′. The PCR products for the deletion (1,323 bp) and the wild-type (4,226 bp) alleles were applied on a 1% agarose gel and were sequenced.

### Statistical Analysis

Continuous variables were reported as median with interquartile range (IQR). Categorical data were compared using the chi-square test. PFS and OS were plotted using the Kaplan–Meier method and compared by log-rank test. A Cox proportional hazard model was used for univariate and multivariate analysis for crizotinib PFS and OS. Variables with *p* < 0.2 in the univariate analysis and clinically important variables such as ALK variant type, BIM deletion, and brain metastasis prior to crizotinib were forced into the final model. Statistical significance was set at *p* < 0.05. All statistical analyses were performed using the Statistical Package for the Social Sciences, version 18.0K (SPSS, Inc., Chicago, IL, USA). The data cutoff date was September 23, 2018.

## Results

### Patient Demographic and Clinical Characteristics

A total of 104 ALK IHC(+) patients received crizotinib for advanced NSCLC during the study period. Fifty-five patients had known EML–ALK fusion variants, as determined by RT-PCR. One patient who received crizotinib for <30 days because of side effects was excluded. A total of 54 patients with known EML4–ALK fusion variants were included in the study. Because of the overlapping enrollment interval, 13 of the 54 patients were included in another published article (31). Thirty of the total 54 patients had adequate tissue for BIM polymorphism analysis.

Twenty-three patients had ALK variant 1; six patients had ALK variant 2; 18 patients had ALK variant 3a/b; and seven patients had other ALK variants (two with variant 5, one with variant V5a, two with variant 6, one with variant 8, and one with variant 1 plus insertion of 117 base pairs). The median follow-up time of the cohort was 13.8 (IQR, 7.4–25.4) months. Most patients had received prior anticancer therapy (median, 2 lines of prior anticancer therapy before crizotinib, range, 0–12) and three patients had received crizotinib as first-line therapy. The crizotinib response rate and the median follow-up time did not differ between the different ALK variant groups (Table 1). Patients with variant 2 had better ECOG performance status (0 or 1) (Table 1). Patients with long ALK variants were younger than patients with short ALK variants (*p* = 0.03) (Supplementary Table 1). In patients with long ALK variants, the baseline characteristics were not different significantly between variant 2 and other long ALK variants (Supplementary Table 2).

**Table 1:** Demographic data (*n* = 54).

**Variable**	**Variant 1 (*n* = 23)**	**Variant 2 (*n* = 6)**	**Variant 3a/b (*n* = 18)**	**Other variants (*n* = 7)**	***p*-value**
Median age (years) (IQR)	56 (47–62)	50 (45–57)	62 (55–65)	56 (46–61)	0.16
Male	13 (57%)	2 (33%)	12 (67%)	5 (71%)	0.46
Never-smoker	13 (57%)	6 (100%)	13 (72%)	6 (86%)	0.14
Previous anticancer therapy (line)	2 (1–5)	1 (1–5)	2 (1–3)	4 (4, 5)	0.15
ECOG ≥2 before crizotinib	5 (22%)	0 (0%)	2 (11%)	4 (57%)	0.04
Brain metastasis before crizotinib	10 (44%)	1 (17%)	5 (28%)	4 (57%)	0.34
Best crizotinib response^*^					0.81
*PR*	9 (43%)	4 (66%)	11 (60%)	4 (57%)	
*SD*	8 (38%)	1 (17%)	4 (22%)	1 (14%)	
*PD*	4 (19%)	1 (17%)	3 (18%)	2 (29%)	
BIM deletion (*n* = 30)	3/12 (25%)	0/4 (0%)	2/9 (22%)	1/5 (20%)	0.75
Median follow-up time (months) (IQR)	15.4 (5.0–22.3)	18.2 (10.2–43.9)	15.9 (8.7–35.2)	9.9 (7.7–20.3)	0.47

* *Two patients with variant 1 were not evaluable for crizotinib response*.

*IQR, interquartile range; ECOG, Eastern Cooperative Oncology Group performance score; BIM, Bcl-2-like 11*.

BIM deletion polymorphism was found in 20% (6/30) of the patients. There was no significant difference in demographic data between patients with deletion polymorphism and wild type (Supplementary Table 3).

### Progression-Free Survival

**Figure 1:**
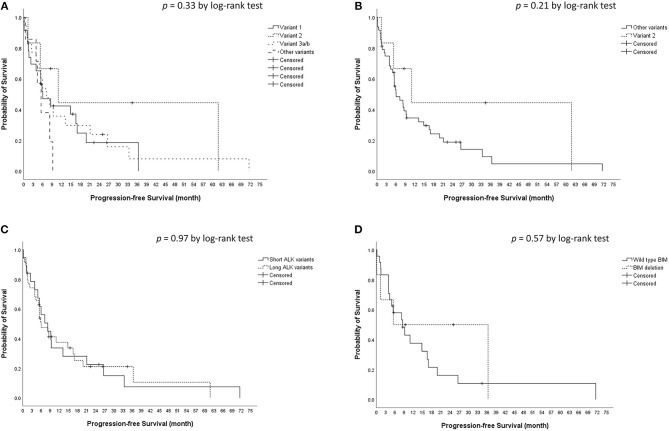
Kaplan–Meier analyses for progression-free survival (PFS). **(A)** PFS among different ALK fusion variants. **(B)** PFS between ALK fusion variant 2 and other fusion variants. **(C)** PFS between short (variant 3a/b and 5a/b) and long (all other variants) ALK fusion variants. **(D)** PFS between BIM deletion and wild type BIM.

The median crizotinib PFS was 7.3 [95% confidence interval (CI), 4.2–10.4] months in this cohort. The median PFS did not differ significantly among the four ALK variant groups [variant 1, 6.1 (95% CI, 1.6–10.6) months; variant 2, 11.0 (95% CI, 0–22.1) months; variant 3, 7.3 (95% CI, 3.6–10.9) months; other variants, 5.5 (95%, 3.1–8.0) months, *p* = 0.33 by log-rank test, Figure 1A]. The median PFS also did not differ significantly between variant 2 and all other variants [variant 2, 11.0 (95% CI, 0–22.1) months; all other variants, 6.1 (95% CI, 2.7–9.5) months, *p* = 0.21 by log-rank test, Figure 1B], between long ALK variants and short variants [long ALK variants, 6.1 (95% CI 2.3–9.8) months; short ALK variants, 8.2 (95% CI, 3.7–12.7) months, *p* = 0.97 by log-rank test, Figure 1C], and between BIM deletions and not [BIM deletion, 5.5 (95% CI 0–26.6) months; wild-type BIM, 8.6 (95% CI, 3.5–13.7) months, *p* = 0.57 by log-rank test, Figure 1D]. Multivariate analysis found that ECOG performance status ≥2 [adjusted hazard ratio (aHR) 8.35, 95% CI, 1.52–45.94, *p* = 0.015] and previous anticancer therapy (aHR 1.35, 95% CI, 1.04–1.76 for each additional line of therapy, *p* = 0.025) were independent factors for crizotinib PFS (Table 2). However, EML4–ALK fusion variants and BIM deletion were not independent factors for crizotinib PFS. ALK variant 1, variant 2, and variant 3a/b had nearly equal aHR (1.00 as the reference, 0.99 and 1.30, respectively). BIM deletion had a nearly neutral aHR 0.88, as well.

**Table 2:** Progression-free survival: univariate and multivariate analysis (*n* = 54).

**Variable**	**Univariate analysis**			**Multivariate analysis**		
	**Hazard ratio**	**95% CI**	***p*-value**	**Adjusted hazard ratio**	**95% CI**	***p*-value**
Age (≥65)	0.79	0.35–1.80	0.58			
Male sex	1.04	0.56–1.93	0.89			
Never-smoker	0.94	0.49–1.81	0.86			
ALK variants			0.36			0.87
Variant 1	1.00^b^			1.00^b^		
Variant 2	0.52	0.17–1.59	0.25	0.99	0.18–5.35	0.99
Variant 3a/b	0.93	0.46–1.86	0.93	1.30	0.38–4.43	0.68
Other variants^a^	1.73	0.67–4.47	0.26	0.64	0.14–2.94	0.57
ECOG ≥2	3.76	1.72–8.21	0.001	8.35	1.52–45.94	0.015
Previous anticancer therapy (per line)	1.14	1.02–1.27	0.02	1.35	1.04–1.76	0.025
Initial brain metastasis	1.53	0.82–2.85	0.18	0.72	0.18–2.87	0.64
BIM deletion	0.73	0.25–2.18	0.58	0.88	0.27–2.86	0.83

a *ALK variants other than variants 1, 2, or 3a/b*.

b *As a reference compared to other ALK variants*.

*CI, confidence interval; ALK, anaplastic lymphoma kinase; ECOG, Eastern Cooperative Oncology Group performance score; BIM, Bcl-2-like 11*.

### Overall Survival

**Figure 2:**
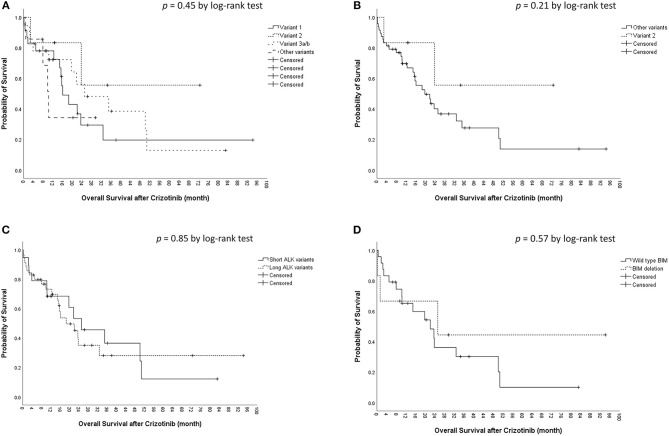
Kaplan–Meier analyses for overall survival (OS). **(A)** OS among different ALK fusion variants. **(B)** OS between ALK fusion variant 2 and other fusion variants. **(C)** OS between short (variant 3a/b and 5a/b) and long (all other variants) ALK fusion variants. **(D)** OS between BIM deletion and wild-type BIM.

The median OS was 22.0 [95% confidence interval (CI), 15.3–28.7) months in the cohort. The median OS did not differ significantly among the four ALK variant groups [variant 1, 16.1 (95% CI, 10.6–21.5) months; variant 2, not reached; variant 3, 25.1 (95% CI, 5.4–44.7) months; other variants, 10.3 (95%, 7.7–12.9) months, *p* = 0.45 by log-rank test, Figure 2A]. The median OS also did not differ significantly between variant 2 and all other variants [variant 2, not reached; all other variants, 19.7 (95% CI, 11.9–27.4) months, *p* = 0.21 by log-rank test, Figure 2B], between long ALK variants and short variants [long ALK variants, 18.5 (95% CI 10.3–26.8) months; short ALK variants, 25.1 (95% CI, 9.2–40.9) months, *p* = 0.85 by log-rank test, Figure 2C], and between BIM deletion and not [BIM deletion, 25.1 (95% CI 0–71.6) months; wild-type BIM, 22.0 (95% CI, 11.1–32.9) months, *p* = 0.57 by log-rank test, Figure 2D]. Multivariate analysis found that ECOG performance status ≥2 (aHR 7.20, 95% CI, 1.27–40.79, *p* = 0.026) was an independent factor for OS (Table 3), while ALK fusion variants and BIM deletion were not.

**Table 3:** Overall survival: univariate and multivariate analysis (*n* = 54).

**Variable**	**Univariate analysis**			**Multivariate analysis**		
	**Hazard ratio**	**95% CI**	***p*-value**	**Adjusted hazard ratio**	**95% CI**	***p*-value**
Age (≥65)	1.07	0.44–2.63	0.89			
Male sex	1.40	0.68–2.90	0.36			
Never-smoker	0.72	0.34–1.51	0.38			
ALK variants			0.48			0.48
Variant 1	1.00^b^			1.00^b^		
Variant 2	0.37	0.08–1.64	0.19	1.15	0.20–6.75	0.88
Variant 3a/b	0.74	0.33–1.64	0.45	1.13	0.28–4.49	0.86
Other variants^a^	1.27	0.41–3.88	0.68	0.22	0.03-1.53	0.13
ECOG ≥2	3.09	1.38–6.93	0.006	7.20	1.27–40.79	0.026
Previous anticancer therapy (per line)	1.15	1.02–1.29	0.028	1.27	0.97–1.66	0.09
Initial brain metastasis	1.65	0.79–3.43	0.18	0.89	0.19–4.13	0.88
BIM deletion	0.70	0.20–2.43	0.57	0.97	0.24–3.95	0.97

a *ALK variants other than variants 1, 2, or 3a/b*.

b *As a reference compared to other ALK variants*.

*CI, confidence interval; ALK, anaplastic lymphoma kinase; ECOG, Eastern Cooperative Oncology Group performance score; BIM, Bcl-2-like 11*.

## Discussion

We found that clinical factors such as prior anticancer therapy and ECOG performance status were independent factors for crizotinib PFS in advanced NSCLC bearing EML4–ALK fusion, while ALK fusion variants and BIM polymorphism were not. In this cohort with mainly previously treated patients, for each additional line of anti-cancer therapy, the adjusted HR was 1.42 (95% CI, 1.06–1.86). With regard to the first-line PROFILE 1014 (2) and second-line PROFILE 1007 (32) phase 3 trials for ALK(+) NSCLC, the median crizotinib PFS in the first-line trial seemed to be better (10.9 vs. 7.0 months). Zhou et al. reported on 73 ALK(+) NSCLC patients that received first-line crizotinib, pemetrexed/platinum, or non-pemetrexed chemotherapy/platinum. Poor ECOG performance status and crizotinib after non-pemetrexed chemotherapy were two independent factors for poor crozitinib PFS in multivariate analysis (33). Lin et al. reported on 94 advanced ALK(+) NSCLC patients and found that crizotinib had a better PFS in first-line use than in second-line use (median PFS 10.5 vs. 8.3 months, *p* = 0.020) (21). Unlike EGFR-TKIs, whose performance was the same in first-line or second-line treatment for advanced EGFR-mutant NSCLC (34), crizotinib had a tendency to do better in first-line use. Poor ECOG performance prior to crizotinib therapy was another independent factor for crizotinib PFS and OS. Poor performance status is a traditional negative prognostic marker among oncology patients (35). It was also found in crizotinib-treated advanced ALK(+) NSCLC patients in previous reports (8, 9, 33, 36).

Different EML4–ALK fusion variants were reported to influence crizotinib efficacy, but results from different reports are conflicting. The results of the current study and of previous reports regarding EML4–ALK fusion variants and crizotinib PFS are summarized in Table 4 (16, 18–23, 37). All studies were conducted in a single center except for the report by Mitiushkina et al., which included three different hospitals in St. Petersburg, Russia (22). Yoshida et al. *first* reported that ALK fusion variant 1 had better crizotinib PFS in 35 Japanese patients (18). This was the first clinical report on the influence of different ALK fusion variants on crizotinib PFS. The patient numbers were relatively small and it only included first-line crizotinib-treated patients. Moreover, Lin et al. reported that 55 patients with variant 1 and variant 3 received first-line crizotinib, and the PFS was similar (23). Li et al. (19), Woo et al. (16), and Christopoulos et al. (20) reported responsiveness of patients from China, South Korea, and Germany to crizotinib in 2018, and the results were similar. Although Li et al. concluded that variant 2 had better crizotinib PFS, there was still a tendency for non-variant 3a/b patients to have a longer crizotinib PFS (median, 18.4 vs. 13.1 months, *p* = 0.24), which was consistent with the findings reported by Woo et al. and Christopoulos et al. The three studies had a similar characteristic: the majority of patients had variant 3a/b. However, while variant 3 had disadvantages in both PFS and OS as reported by Christopoulos et al. (20), the OS was almost the same (*p* = 0.96), as reported by Woo et al. (16). On the other hand, the largest cohort to date by Lin et al. showed that there was no difference between variant 1 and variant 3, in patients treated with both first-line ALK TKI as crizotinib and first-line crizotinib (23). Lei et al. (21), Cha et al. (37), Mitiushkina et al. (22), and our study found no difference between different ALK fusion variants. In the five studies from the United States, China, South Korea, Russia, and Taiwan, the majority of patients had variant 1. Both positive and negative reports included Caucasian and Asian patients, so race may not have contributed to the differences in results. Is it possible that the composite of variants in study cohorts had some influence on the results? The patient percentages of variant 3 in the five studies, which did not find differences between variants, were 48%, 30%, 19%, 25%, and 33%, respectively. In fact, patients with variant 3 were the second largest group among the cohorts, which does not lend support to the hypothesis of smaller patient numbers leading to an overestimation of PFS for variant 3 in the studies. One of the possible explanations may be the use of multivariate analysis. Only studies by Yoshida et al. and Li et al., and our study used a multivariate analysis to determine the independent factors for crizotinib PFS. Although the clinicopathologic characteristics seemed to be similar between the two analyzed groups (such as variant 3a/b or non-variant 3a/b), multivariate analysis that includes clinically relevant variables may still be a better method to find independent factors. Different patient groupings may influence crizotinib PFS if they are not adjusted appropriately. This may partly explain the discordance of OS data between the reports from Woo et al. and Christopoulos et al. In Christopoulos's cohort, variant 3a/b patients had more initial metastatic sites, either thoracic or extra-thoracic, and fewer patients with variant 3a/b had cancer recurrence from an early-stage cancer rather than initial stage IV (20). More metastatic sites and less cancer recurrence from early-stage NSCLC had survival disadvantages (38, 39), and might have contributed to shorter PFS and OS in variant 3a/b patients in the cohort. On the other hand, in our cohort, although patients with variant 2 tended to have a longer PFS, they might also have clinical advantages (tended to be younger, never-smokers, with better baseline performance status, and with less initial brain metastasis) (Table 1). The PFS between variant 1, variant 2, and variant 3a/b were almost equal after multivariate analysis (aHR, 1.00 as reference, 0.99, 1.30, respectively, Table 2). We hypothesize that although different ALK fusion variants might contribute to different crizotinib PFS, the impact may not be significant after adjusting for clinical factors.

**Table 4:** Current reports of ALK fusion variants and crizotinib progression-free survival.

**References**	**Study site**	**Detection of ALK fusion**	**ALK TKI**	**Timing of ALK TKI**	**Patient number**	**Prominent ALK fusion variant**	**Non-EML4-ALK fusion**	**PFS difference**	**Median ALK TKI PFS (months)**	**Multi-variate analysis**
Yoshida et al. (18)	Japan	RT-PCR	Crizotinib	First-line	35	**V1, 54%** (54%/14%/12%)^*^	No	V1 longer	11.0 vs. 4.2	Yes
Li et al. (19)	China	NGS	Crizotinib	Mixed	49	**V3a/b, 33%** (23%/15%/33%)^*^	Yes (18%)	V2 longer	34.5 vs. 12.3	Yes
Woo et al. (16)	South Korea	RT-PCR	Crizotinib	Mixed	44	**V3a/b, 44%** (33%/11%/44%)^*^	Yes (6%)	Non-V3a/b longer	Not-reached vs. 11.0	No
Christopoulos et al. (20)	Germany	RT-PCR, NGS	Crizotinib, Alectinib, Ceritinib	Mixed	67	**V3a/b, 51%** (39%/10%/51%)^*^	No	Non-V3a/b Longer	39.3 vs. 7.3	No
Lin et al.^†^ (23)	United States	RT-PCR, DNA direct sequencing or NGS	Crizotinib	First-line ALK TKI^a^ and first-line^b^	99^†*a*^ 55^b^	**V1**^†^ **52%** (V3 48%)^**a**^ **V3**^†^ **51%** (V1 49%)^**b**^	No	No difference^a^^,^^b^	9.2 vs. 7.5^a^ 8.9 vs. 6.9^b^	No
Lei et al. (21)	China	RACE-coupled PCR	Crizotinib	Mixed	61	**V1, 36%** (36%/12%/30%)^*^	Yes (3%)	No difference	V1 vs. V3 vs. others: 11 vs. 10.9 vs. 7.4	No
Cha et al. (37)	South Korea	RT-PCR	Crizotinib	Mixed	32	**V1, 39%** (39%/6%/19%)^*^	Yes (37%)	No difference	Not disclosed in numbers^#^	No
Mitiushkin et al. (22)	Russia	RT-PCR	Crizotinib, Alectinib, Ceritinib	Mixed	64	**V1, 52%** (52%/5%/25%)^*^	Yes (2%)	No difference	Not disclosed in numbers^#^	No
Current study	Taiwan	RT-PCR	Crizotinib	Mixed	54	**V1, 43%** (43%/11%/33%)^*^	No	No difference	V1 vs. V2 vs. V3 vs. others: 6.1 vs. 11.0 vs. 7.3 vs. 5.5	Yes

* *Proportion of variant 1/variant 2/variant 3a/b in study cohorts*.

† It is the largest cohort to date. It only compared variant 1 with variant 3. Data from patients with other ALK variants were not disclosed. Patients who received crizotinib as first-line ALK TKI

a and crizotinib as first-line therapy

b *were listed*.

# *Only Kaplan–Meier curves were available*.

*The bold values were used to emphasize the prominent ALK fusion variant and its percentage only*.

In this study, there were 30 patients with enough tissue for BIM analysis and six were positive for BIM deletion (20%). The prevalence rate was consistent with previous reports (11–19%) (24–27). The BIM deletion polymorphism was not associated with a difference in crizotinib PFS (Figure 1D) or OS (Figure 2D). Using the multivariate Cox proportional hazard model, BIM deletion was also not related to differences in PFS or OS (Tables 2, 3). BIM deletion was associated with shorter PFS in 47 ALK(+) NSCLC patients receiving crizotinib (26). BIM polymorphism was also reported to be associated with primary resistance or short PFS with EGFR TKIs (24, 25). However, Lee et al. checked 193 patients who received EGFR TKI for EGFR-mutant NSCLC and there was also no difference in EGFR TKI PFS between patients with and those without a BIM deletion (40). The result was similar to our previous analysis (27). Although BIM is a pro-apoptotic protein and may be related to TKI-induced cancer cell death, lung cancer cells may not be totally dependent on this pathway, and the concentration of BIM protein may also matter. Furthermore, the BIM deletion polymorphism is found only in Asians, and not in Caucasians (24). If the BIM deletion polymorphism was associated with shorter PFS, the effectiveness of crizotinib among Asians would be worse than in Western countries, but this is not true. Whether or not a simple BIM gene deletion influences TKI efficacy in NSCLC patients remains questionable.

There were several limitations to this study. First, it was a retrospective cohort study in a single center, as in previous reports. Because of the rarity of ALK(+) NSCLC, the patient number was still limited. The BIM deletion polymorphism in ALK(+) NSCLC patients, which is found in only 10–20% of ALK(+) patients, is even rarer. This may also be the reason that different reports have had different findings to date. As a result of limited patient numbers, the resistance mechanisms could not be addressed. Further larger multicenter or international prospective cohorts are warranted. Second, this was a cohort with mainly previously treated patients. Our results may not be generalizable to patients receiving first-line crizotinib therapy. Because reimbursement of crizotinib as first-line therapy was not approved by Taiwan's National Health Insurance until November 1, 2017, only three of 54 patients in our cohort used crizotinib as first-line therapy. However, although the U.S. FDA approved first-line crizotinib therapy, almost other studies also included mixed-line therapy with crizotinib, and purely first-line crizotinib data were rare (Table 4). Third, we used RT-PCR to determine ALK fusion variants. As in prior reports, not all ALK fusion variants could be detected. With the development of next-generation sequencing, more ALK fusion variants can be found, and the entire picture of ALK fusion lung cancer will become clearer.

In conclusion, clinical factors such as more prior anticancer therapies and ECOG performance status ≥2 were associated with a poorer crizotinib outcome. Different ALK variants and the BIM polymorphism were not independent factors for crizotinib PFS or OS in this study.

## Data Availability

The datasets that were analyzed during the current study are available from the corresponding author on reasonable request.

## Ethics Statement

This study was reviewed and approved by the Institutional Review Board of National Taiwan University Hospital. Written informed consent was obtained from all patients before checking their cancer specimens for molecular studies. All methods were performed in accordance with the relevant guidelines and regulations.

## Author's Note

An earlier version of this study was presented as a poster presentation in the Asian Pacific Society of Respirology 2018 Congress.

## Author Contributions

Y-TL participated in the study design, review and collection of patients' data, statistical analysis, and drafting of the manuscript. Y-NL participated in collection of patients' data, analyses of ALK and BIM, and revision of the manuscript. J-YS designed the study, interpreted the data, and reviewed and revised the manuscript.

### Conflict of Interest Statement

Y-TL has received speaking honoraria from AstraZeneca, Boehringer Ingelheim, Bristol-Myers Squibb, Pfizer, Roche, and TTY Biopharm; and travel expense from Pfizer. J-YS has received personal fees for advisory boards from AstraZeneca, Roche, Boehringer Ingelheim, Eli Lilly, Merck Sharp & Dohme, Ono Pharmaceutical, Chugai Pharmaceutical, AbbVie, and Bristol-Myers Squibb; speaking honoraria from AstraZeneca, Roche, Boehringer Ingelheim, Eli Lilly, Pfizer, Novartis, Merck Sharp & Dohme, Ono Pharmaceutical, Chugai Pharmaceutical, AbbVie, and Bristol-Myers Squibb; and travel expense from Roche, Boehringer Ingelheim, Pfizer, Merck Sharp & Dohme, Chugai Pharmaceutical, and Bristol-Myers Squibb. The remaining author declares that the research was conducted in the absence of any commercial or financial relationships that could be construed as a potential conflict of interest.
