# Supplementary material for: The Impact of Clinical Factors, ALK Fusion Variants, and BIM Polymorphism on Crizotinib-Treated Advanced EML4–ALK Rearranged Non-small Cell Lung Cancer
Source: Front Oncol. 2019 Sep 23;9:880. doi: 10.3389/fonc.2019.00880 (PMC6768009; doi:10.3389/fonc.2019.00880)
Supplement: Supplementary file 1 [file Data_Sheet_1.docx]

| **Supplementary Table 1. Demographic Data Stratified by Long and Short ALK Variants (n=54).** | | | | |
| --- | --- | --- | --- | --- |
| Variable | Long Variants^1^  (n=35) | Short Variants^2^ (n=19) | *p* value |  |
| **Median age (year-old) (IQR)** | 55 (47-61) | 61 (55-64) | 0.03 |  |
| **Male** | 19 (54%) | 13 (68%) | 0.31 |  |
| **Never smoker** | 24 (69%) | 14 (74%) | 0.69 |  |
| **Previous anticancer therapy (line) (IQR)** | 2 (1-5) | 2 (1-4) | 0.30 |  |
| **ECOG ≥ 2 before crizotinib** | 9 (26%) | 2 (11%) | 0.19 |  |
| **Brain metastasis before crizotinib** | 15 (43%) | 5 (26%) | 0.23 |  |
| **Best crizotinib response**^3^ |  |  | 0.87 |  |
| *PR* | 17 (52%) | 11 (58%) |  |  |
| *SD* | 9 (27%) | 5 (26%) |  |  |
| *PD* | 7 (21%) | 3 (16%) |  |  |
| **BIM deletion (n=30)** | 4/20 (20%) | 2/10 (20%) | 0.99 |  |
| **Median follow up time (month) (IQR)** | 14.8 (6.5 – 23.5) | 12.1 (10.3 – 34.9) | 0.53 |  |
|  |  |  |  |  |
| ^1^ Long ALK variants: Variants other than short ALK variants  ^2^ Short ALK variants: Variant 3a, Variant 3b, Variant 5a, Variant 5b  ^3^ Two patients with variant 1 were not evaluable for crizotinib response.  Acronyms: ALK, anaplastic lymphoma kinase; IQR, interquartile range; ECOG, Eastern Cooperative Oncology Group performance score; PR, partial response; SD, stable disease; PD, progressive disease; BIM, Bcl-2-like 11 | | | | |

| **Supplementary Table 2. Demographic Data in Patients with Long ALK Variants^1^ (n=35).** | | | | |
| --- | --- | --- | --- | --- |
| Variable | Variant 2  (n=6) | Other long variants (n=29) | *p* value |  |
| **Median age (year-old) (IQR)** | 50 (45-57) | 56 (47-62) | 0.37 |  |
| **Male** | 2 (33%) | 17 (59%) | 0.26 |  |
| **Never smoker** | 6 (100%) | 18 (62%) | 0.07 |  |
| **Previous anticancer therapy (line) (IQR)** | 1 (1-5) | 3 (2-5) | 0.21 |  |
| **ECOG ≥ 2 before crizotinib** | 0 (0%) | 9 (31%) | 0.11 |  |
| **Brain metastasis before crizotinib** | 1 (17%) | 14 (48%) | 0.15 |  |
| **Best crizotinib response**^2^ |  |  | 0.70 |  |
| *PR* | 4 (66%) | 13 (48%) |  |  |
| *SD* | 1 (17%) | 8 (30%) |  |  |
| *PD* | 1 (17%) | 6 (22%) |  |  |
| **BIM deletion (n=30)** | 0/4 (0%) | 4/16 (25%) | 0.26 |  |
| **Median follow up time (month) (IQR)** | 18.2 (10.2-43.9) | 14.8 (5.7 – 22.1) | 0.27 |  |
|  |  |  |  |  |
| ^1^ Long ALK variants: Variants other than Variant 3a, Variant 3b, Variant 5a, Variant 5b  ^2^Two patients with variant 1 were not evaluable for crizotinib response.  Abbreviation: IQR, interquatile range; ALK, anaplastic lymphoma kinase; ECOG, Eastern Cooperative Oncology Group performance score; BIM, Bcl-2-like 11 | | | | |

| **Supplementary Table 3. Demographic Data Stratified by BIM Polymorphism (n=30).** | | | | |
| --- | --- | --- | --- | --- |
| Variable | BIM deletion (n=6) | Wild type BIM (n=24) | *p* value |  |
| **Median age (year-old) (IQR)** | 57 (46-66) | 56 (49-66) | 0.86 |  |
| **Male** | 5 (83%) | 14 (58%) | 0.26 |  |
| **Never smoker** | 3 (50%) | 17 (71%) | 0.33 |  |
| **Previous anticancer therapy (line) (IQR)** | 4 (2-6) | 4 (2-5) | 0.98 |  |
| **ECOG ≥ 2 before crizotinib** | 2 (33%) | 5 (21%) | 0.52 |  |
| **Brain metastasis before crizotinib** | 1 (17%) | 9 (38%) | 0.33 |  |
| **ALK fusion variant** |  |  | 0.75 |  |
| Variant 1 | 3 (50%) | 9 (37%) |  |  |
| Variant 2 | 0 (0%) | 4 (17%) |  |  |
| Variant 3 | 2 (33%) | 7 (29%) |  |  |
| Other variants | 1 (17%) | 4 (17%) |  |  |
| **Best crizotinib response**^1^ |  |  | 0.21 |  |
| *PR* | 4 (80%) | 10 (41%) |  |  |
| *SD* | 0 (0%) | 9 (38%) |  |  |
| *PD* | 1 (20%) | 5 (21%) |  |  |
| **Median follow up time (month) (IQR)** | 17.3 (1.0 – 45.6) | 13.8 (6.8 – 30.4) | 0.88 |  |
|  |  |  |  |  |
| ^1^ One patient with BIM deletion was not evaluable for crizotinib response.  Acronyms: BIM, Bcl-2-like 11; IQR, interquartile range; ECOG, Eastern Cooperative Oncology Group performance score; ALK, anaplastic lymphoma kinase; PR, partial response; SD, stable disease; PD, progressive disease | | | | |
